# The SIRT1 Modulators AROS and DBC1 Regulate HSF1 Activity and the Heat Shock Response

**DOI:** 10.1371/journal.pone.0054364

**Published:** 2013-01-18

**Authors:** Rachel Raynes, Kathleen M. Pombier, Kevin Nguyen, Jessica Brunquell, Jamie E. Mendez, Sandy D. Westerheide

**Affiliations:** The Department of Cell Biology, Microbiology and Molecular Biology, College of Arts and Sciences, University of South Florida, Tampa, Florida, United States of America; Semmelweis University, Hungary

## Abstract

The heat shock response, the cellular response to protein damaging stress, is critical in maintaining proteostasis. The heat shock response is regulated by the transcription factor HSF1, which is activated upon heat shock and other stresses to induce the expression of molecular chaperones. SIRT1 has previously been shown to activate HSF1 by deacetylating it, leading to increased DNA binding ability. We have investigated how the heat shock response may be controlled by factors influencing SIRT1 activity. We found that heat shock results in an increase in the cellular NAD^+^/NADH ratio and an increase in recruitment of SIRT1 to the *hsp70* promoter. Furthermore, we found that the SIRT1 modulators AROS and DBC1 have an impact on *hsp70* transcription, HSF1 acetylation status, and HSF1 recruitment to the *hsp70* promoter. Therefore, AROS and DBC1 are now two new targets available for therapeutic regulation of the heat shock response.

## Introduction

The heat shock response (HSR) is a cellular response to diverse stressors that results in the induction of genes encoding molecular chaperones essential for protection and recovery from cellular damage [1]. The stressors that activate transcription of these heat shock genes include elevated temperature, heavy metals, chemical toxicants, and oxidative stress. Heat shock proteins function to guide protein folding and protect cells against a wide array of stress conditions [2]. The HSR is regulated at the transcriptional level by the heat shock transcription factor 1 (HSF1).

SIRT1 is a NAD^+^-dependent deacetylase that regulates a number of target proteins, including p53 [3], [4], PGC-1α [5], [6], HIV Tat [7], [8], NF-κB [9], [10], [11], and the FOXO family of transcription factors [12], [13], [14]. We have previously shown that SIRT1 also impacts the HSR by deacetylation of the transcription factor HSF1 [15]. However, the mechanism by which heat shock (HS) may regulate SIRT1 is still unknown.

SIRT1 has been shown to be regulated by diverse mechanisms. SIRT1 mRNA levels are stabilized by HuR, a ubiquitously expressed RNA binding protein, and oxidative stress has been reported to dissociate the *HuR-sirt1* mRNA complex allowing for *sirt1* mRNA decay [16]. SIRT1 enzymatic activity can be regulated by the cellular NAD^+^/NADH ratio, as the hydrolysis of NAD^+^ is necessary to drive SIRT1 enzymatic activity [17]. Caloric restriction (CR) was shown to increase the replicative lifespan of yeast by activating Sir2, the yeast SIRT1 homolog, due to decreasing NADH levels [18]. Interestingly, CR induces the expression of NAMPT, which triggers the NAD^+^ salvage pathway converting nicotinamide (NAM) to NAD^+^ [19].

Recently, DBC1 and AROS have been indicated to have a role in SIRT1 regulation. DBC1, identified as a SIRT1 interactor via co-purification, was found to inhibit the deacetylase activity of SIRT1, resulting in increased p53 acetylation and the upregulation of p53 function and p21 gene expression [20], [21]. Conversely, AROS, identified as a SIRT1 interactor via a yeast two-hybrid screen, was found to activate SIRT1, promoting the deacetylation of p53 and leading to the downregulation of p53 function [22]. The impact of SIRT1 expression levels, changes in the cellular NAD^+^/NADH ratio, and the SIRT1 modulators DBC1 and AROS have yet to be investigated as they relate to the regulation of the HSR.

Here we further investigate the mechanism of SIRT1 regulation of the HSR. Previously, HSF1 has been shown to interact with SIRT1 via co-immunoprecipitation, indicating that HSF1 and SIRT1 interact, but not indicating whether or not the interaction is direct [15], [23]. We now show with a GST pull-down assay that the HSF1-SIRT1 interaction is direct. We report that induction of the HSR correlates with an increase in the cellular NAD^+^/NADH ratio and with an increase in recruitment of SIRT1 to the *hsp70* promoter. Additionally, we find that the SIRT1 modulators AROS and DBC1 have an impact on *hsp70* transcription, HSF1 acetylation status, and HSF1 recruitment to the *hsp70* promoter.

## Results

### SIRT1 is not Regulated by a Change in Expression upon HS

**Figure 1:**
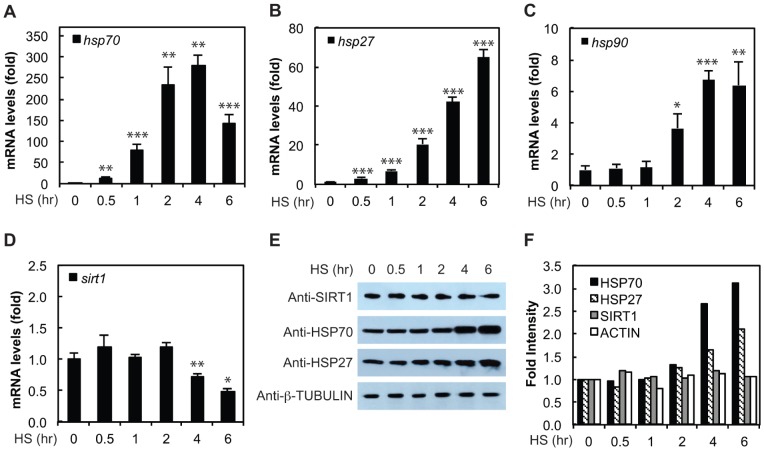
SIRT1 is not regulated by a change in expression upon HS. (A–C) *hsp70*, *hsp27,* and *hsp90* mRNA expression levels are induced during a HS timecourse. HeLa cells were exposed to a 42°C HS from 0 to 6 hours and mRNA levels were determined by qRT-PCR. (D) *sirt1* mRNA expression levels are not altered in the early stages of HS. HeLa cells were exposed to a 42°C HS from 0 to 6 hours and mRNA levels were determined qRT-PCR. Results for A–D are in technical triplicates and are representative of biological duplicates. Statistical significance was measured by Student’s t test as compared to 0 hr HS (*P<0.05; ** *P<0.01).* (E) SIRT1 protein levels are not altered during a HS timecourse. HeLa cells were treated with a HS timecourse and SIRT1, HSP70, HSP27, and β-Tubulin protein levels were determined by Western analysis. (F) Protein levels from E were quantified and plotted using ImageJ.

We sought to determine if HS might regulate the activity of SIRT1 through changes in SIRT1 expression levels. HeLa cells were treated with a 42°C HS from 0 to 6 hours and, as expected, *hsp70* mRNA levels increased substantially by 2 hours of HS and then began to decline by 6 hours of treatment correlating with the attenuation phase of the HSR (Fig. 1A). The mRNA levels for *hp27* and *hsp90* were also quantified, demonstrating that HS induces the transcription of multiple HSPs (Fig. 1B, C). Conversely, *sirt1* mRNA levels were not altered during the initial 2 hours of HS treatment (Fig. 1D). While *sirt1* mRNA levels did begin to decline by 4 hours of HS treatment, Western blot analysis shows that SIRT1 protein levels remained constant throughout the 6 hour HS time course, while HSP70 and HSP27 protein levels were increased at the 4 and 6 hour timepoints as expected (Fig. 1E, F). HSP90 protein levels did not show a significant increase over this timecourse, likely due to the abundance and stability of this protein (data not shown) [24]. Therefore, we conclude that the regulation of SIRT1 abundance is likely not a major mechanism by which HS controls SIRT1 activity, at least during the initial phases of the HSR.

### Heat Shock Induces an Increase in the Cellular NAD^+^/NADH Ratio

**Figure 2:**
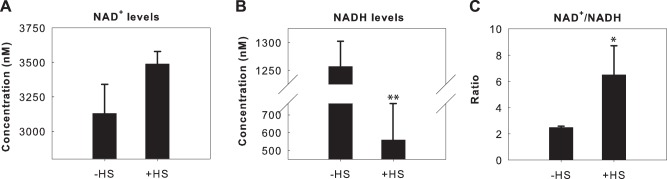
HS induces an increase in the NAD^+^/NADH ratio. (A) NAD^+^ and (B) NADH concentrations were determined for HeLa cells exposed to either control conditions or a 42°C HS for 2 hours. (C) HS induces an increase in the NAD^+^/NADH ratio as a result of decreased NADH levels. Mean values of the NAD^+^/NADH ratios from (A) and (B) are plotted. Results in A-C are in technical triplicates and are representative of biological duplicates. Statistical significance was measured by Student’s t test as compared to - HS (*P<0.05; ** *P<0.01;* ***P<0.001).

We next investigated whether HS could induce a change in the cellular NAD^+^/NADH ratio, as SIRT1 is well-documented as an NAD^+^-dependent deacetylase [25], [26], [27], [28]. The cellular NAD^+^ and NADH levels from whole cell extracts were quantified for HEK293 cells that were treated with HS at 42°C for 2 hours and the levels compared to unstressed cells. HS did not induce a significant increase in the cellular NAD^+^ levels (Fig. 2A), but did significantly decrease the cellular NADH levels by approximately 2-fold (Fig. 2B). Overall, HS resulted in an increase of the NAD^+^/NADH ratio from 2.5 to 6.5 (Fig. 2C). HS may therefore have a positive impact on SIRT1 activity due to an increase in available NAD^+^ and a reduction of NADH. We note that as SIRT1 is a nuclear protein, a more relevant measurement may be of the nuclear (instead of the whole cell) NAD^+^/NADH ratio. Future studies will be required to determine if the nuclear NAD^+^/NADH ratio is altered upon HS.

### SIRT1 Recruitment to the hsp70 Promoter Increases upon Heat Shock

**Figure 3:**
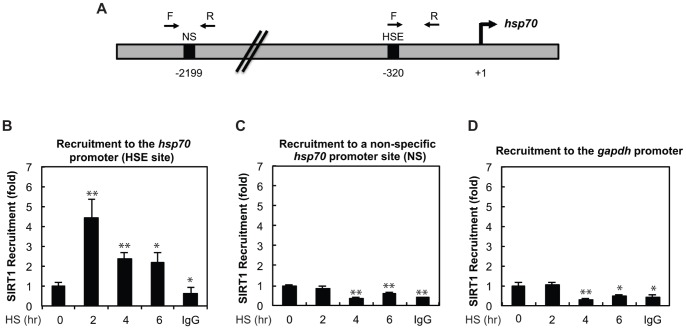
HS results in an increase in SIRT1 recruitment that is specific to the HSE within the *hsp70* promoter. (A) Schematic representation of primers designed to amplify the HSE site and a nonspecific (NS) site at the *hsp70* promoter. (B) SIRT1 is recruited to the *hsp70* promoter HSE site during HS. HEK293 cells were exposed to a 42°C HS from 0 to 6 hours prior to chromatin immunoprecipitation (ChIP) with a SIRT1 antibody. SIRT1 is not recruited upon HS to a non-specific (NS) upstream *hsp70* promoter site (C) or to the *gapdh* promoter (D). Results in B-D are in technical triplicates and are representative of biological duplicates. Statistical significance was measured by Student’s t test as compared to - HS (*P<0.05; ** *P<0.01;* ***P<0.001).

We then tested SIRT1 recruitment to the *hsp70* promoter over a HS time course to see if SIRT1 may be recruited to DNA with the same kinetics as HSF1. HEK293 cells were treated with HS at 42°C for 0, 2, 4, and 6 hours followed by chromatin immunoprecipitation (ChIP) using an antibody to SIRT1. We found that upon a 2 hour HS, there was a 4-fold increase of SIRT1 recruitment to the *hsp70* promoter (Fig. 3B). After 6 hours of HS, recruitment of SIRT1 to the *hsp70* promoter was reduced to a 2-fold increase as compared to unstressed cells. These kinetics correspond to what has previously been reported for HSF1 DNA recruitment [15]. Therefore, it is likely that SIRT1 has the ability to regulate HSF1 while it is on the promoter. Recruitment was also evaluated at an upstream non-specific site of the *hsp70* promoter and at the *gapdh* promoter. We found that SIRT1 recruitment to these sites do not increase upon HS (Fig. 3C, D). Therefore, increased recruitment of SIRT1 is specific to the HSE-binding site within the *hsp70* promoter.

### AROS and DBC1 Expression have an Impact on the HSR

**Figure 4:**
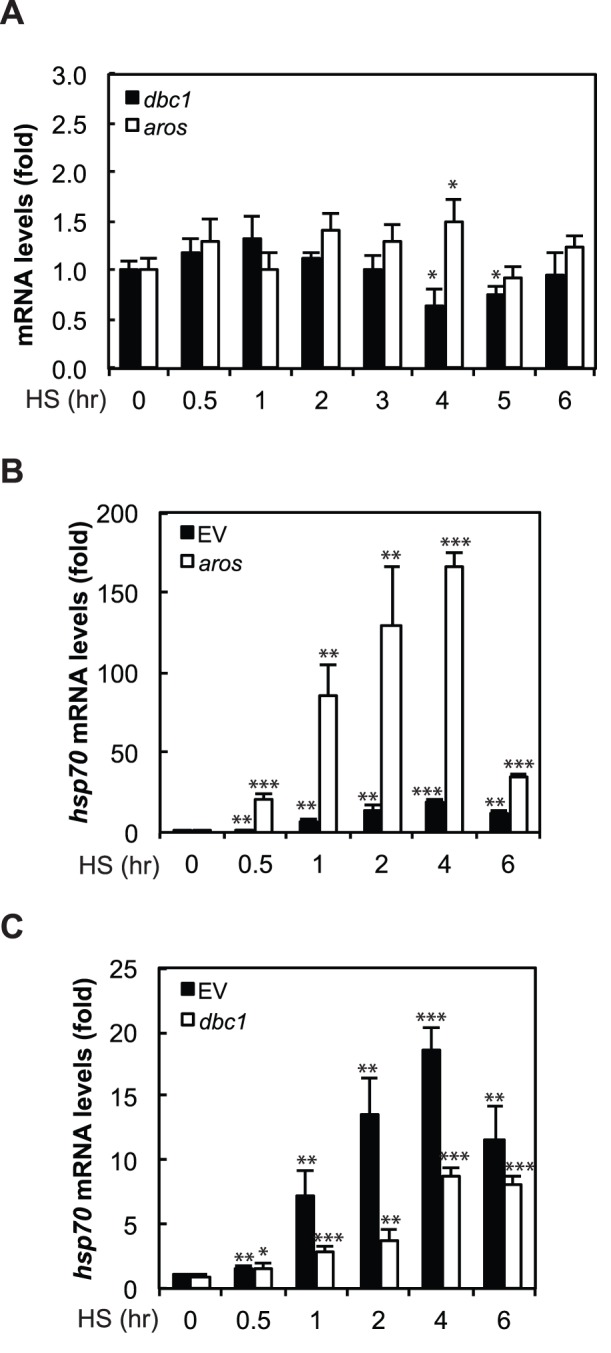
AROS and DBC1 overexpression impacts the transcription of *hsp70* and initiation of the HSR. (A) *aros* and *dbc1* mRNA expression levels do not undergo a significant change upon 0–3 hours of HS, while small changes in levels are observed between 3–6 hours of HS. HEK293 cells were exposed to a 42°C HS from 0 to 6 hours and mRNA levels were determined by qRT-PCR. (B) AROS overexpression enhances HS induction of *hsp70* mRNA, while (C) DBC1 overexpression inhibits HS induction of *hsp70* mRNA. For B and C, HEK293 cells were transfected with empty vector (EV), AROS, or DBC1 and then exposed to a 42°C HS from 0 to 6 hours. The mRNA levels were determined for *hsp70* by qRT-PCR. Results in A-C are in technical triplicates and are representative of biological duplicates. Statistical significance was measured by Student’s t test as compared to 0 hr HS (*P<0.05; **P<0.01; ***P<0.001).

As SIRT1 activity is positively regulated by AROS and negatively regulated by DBC1, we next sought to determine the impact of these proteins on the HSR. While *aros* and *dbc1* mRNA levels do not change upon 0–3 hours of HS and show only minimal change between 3–6 hours of HS (Fig. 4A), AROS and DBC1 overexpression have significant effects on *hsp70* transcription (Fig. 4B, C). AROS, DBC1, or empty vector was overexpressed in HEK293 cells and the induction of the HSR was assessed using the transcription of *hsp70* as a marker throughout a 6 hour HS time course. We found that AROS overexpression resulted in a striking increase in HS-induced *hsp70* mRNA levels (Fig. 4B). Conversely, DBC1 overexpression resulted in a decrease in HS-induced *hsp70* mRNA levels (Fig. 4C).

**Figure 5:**
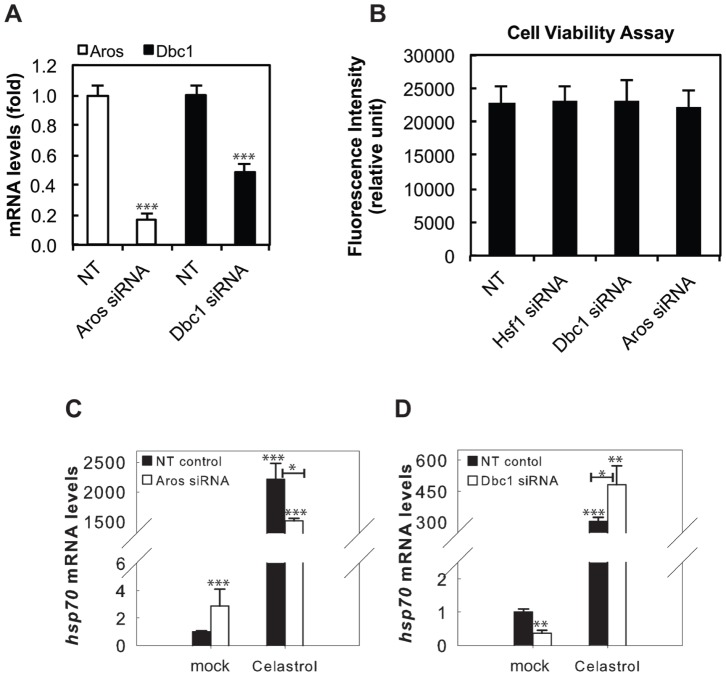
AROS and DBC1 knockdown impact the transcription of *hsp70* and initiation of the HSR. (A) AROS and DBC1 siRNA is able to knockdown gene expression. HEK293 cells were transfected with siRNA for DBC1, AROS, or a non-targeting (NT) control and *aros* and *dbc1* gene expression was assessed by qRT-PCR. (B) AROS, DBC1, and HSF1 siRNA do not significantly impact cell viability compared to the NT siRNA control as measured by a PrestoBlue assay. Cell viability assay was performed in biological and technical triplicates and statistical significance was measured by Student’s t test compared to NT siRNA control. (C) AROS knockdown decreases the induction of *hsp70* by celastrol. HEK293 cells were transfected with AROS or NT control siRNA and treated with and without celastrol (5 µM). The mRNA levels were determined for *hsp70* by qRT-PCR. D) DBC1 knockdown increases the induction of *hsp70* by celastrol. HEK293 cells were transfected with DBC1 or NT control siRNA and treated with and without celastrol (5 µM). The mRNA levels were determined for *hsp70* by qRT-PCR. Results in A, C, and D are in technical triplicates and are representative of biological duplicates. Statistical significance was measured by Student’s t test as compared to control (*P<0.05; ** *P<0.01;* ***P<0.001).

Next, we investigated the impact of AROS and DBC1 siRNA knockdown on the HSR. We transfected HEK293 cells with AROS, DBC1, or a non-targeting (NT) siRNA and treated the cells with either DMSO (mock) or celastrol (5 µM), a potent inducer of the HSR [29]. Transfection with siRNA resulted in significant knockdown of *aros* and *dbc1,* which did not impact cell viability compared to the NT control (Fig. 5A, B). Cells transfected with AROS siRNA displayed a decrease in *hsp70* induction by celastrol (Fig. 5C), while conversely cells transfected with DBC1 siRNA displayed an increase in *hsp70* induction by celastrol (Fig. 5D). Therefore, we conclude from these experiments that AROS and DBC1 may be significant regulators of the HSR, with AROS having a positive effect on the HSR and DBC1 having a negative effect.

### AROS and DBC1 Affect HSF1 Acetylation

**Figure 6:**
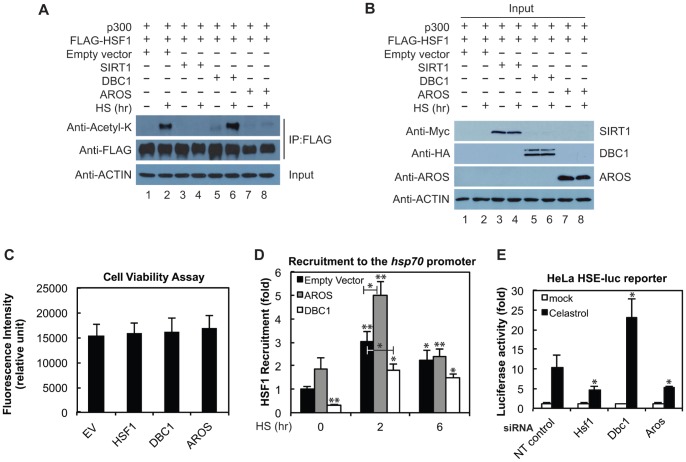
AROS and DBC1 impact HSF1 acetylation, DNA recruitment, and activity at the *hsp70* promoter. (A) AROS and DBC1 overexpression impact HSF1 acetylation. HEK293 cells were transfected with p300 and FLAG-HSF1 as well as empty vector, SIRT1-MYC, DBC1-HA, or AROS as indicated and exposed to 0 or 2 hours of HS at 42°C. A FLAG immunoprecipitation was run on an SDS-PAGE gel and the acetylation status of HSF1 was determined using an acetyl-lysine antibody. Results are representative of biological triplicates. (B) The expression of transfected SIRT1-MYC, DBC1-HA, and AROS was verified by Western analysis. (C) Overexpression of HSF1, DBC1, and AROS do not significantly impact cell viability compared to the empty vector (EV) control as measured by PrestoBlue. The cell viability assay was performed in biological and technical triplicates and statistical significance was measured by Student’s t test compared to EV. (D) AROS and DBC1 overexpression impact HSF1 recruitment to the *hsp70* promoter. AROS, DBC1, or empty vector (EV) was overexpressed in HEK293 cells prior to HS for 0, 2, or 6 hours and chromatin immunoprecipitation (ChIP) was performed with an HSF1 antibody. The purified DNA was then analyzed by qPCR and DNA levels were determined for the *hsp70* promoter. The qPCR results are in technical triplicates and statistical significance was measured by Student’s t test as compared to EV at 0 hr HS (*P<0.0; ** *P<0.01).* ChIP was performed in biological duplicates. (E) AROS and DBC1 knockdown impact *hsp70.1* promoter-luciferase reporter activity. HeLa *hsp70.1* promoter-luciferase reporter cells were transfected with 50 nM of Dharmacon SmartPool DBC1, AROS, HSF1, or non-targeting (NT) control siRNA and HS was induced with celastrol (5 µM). Luciferase activity was measured and compared to that of the NT control. Luciferase assays were performed in biological triplicate. Statistical significance was measured by Student’s t test as compared to the NT siRNA control with celastrol (5 µM) treatment (*P<0.05).

We next examined the impact of AROS and DBC1 on HSF1 acetylation status using an *in vivo* acetylation assay. Cells were transfected with Flag-HSF1 and p300 together with SIRT1, DBC1, AROS, or empty vector expression plasmids prior to treatment with or without HS (Fig. 6A). HS led to HSF1 acetylation (lane 2), while the overexpression of SIRT1 inhibited HS-induced HSF1 acetylation (lane 4), consistent with previous results [15]. Interestingly, the overexpression of DBC1, the SIRT1 inhibitor, enhanced HSF1 acetylation under both non-stress and stress conditions (lanes 5 and 6), while the overexpression of the SIRT1 activator AROS reduced HS-induced HSF1 acetylation to a similar degree as SIRT1 itself (compare lanes 4 and 8). Western blot analysis was used to verify the overexpression of SIRT1, DBC1 and AROS (Fig. 6B), and cell viability results show that the overexpression of these factors does not affect overall cellular fitness (Fig. 6C). We therefore conclude that AROS and DBC1 can modulate the acetylation status of HSF1. We would like to point out that while it is likely that the effects of AROS and DBC1 on HSF1 deacetylation are occurring through SIRT1, we have not demonstrated this here and it could be possible that other deacetylases are involved.

### AROS and DBC1 Impact HSF1 DNA Binding

As HSF1 acetylation status has previously been shown to affect DNA binding ability [15], we investigated the effect of AROS and DBC1 expression on HSF1 binding to the *hsp70* promoter. HEK293 cells were transfected with AROS, DBC1, or empty vector and then treated with HS over a 6 hour timecourse followed by ChIP using an antibody to HSF1. As expected, a 2 hour HS resulted in recruitment of HSF1 to the *hsp70* promoter, and this binding decreased by 6 hours of continuous HS (Fig. 6D). AROS overexpression enhanced HS-induced recruitment of HSF1 to the *hsp70* promoter. Conversely, DBC1 overexpression led to the inhibition of HS-induced HSF1 recruitment. Thus, AROS and DBC1 can modulate both HSF1 acetylation status as well as HS-induced binding of HSF1 to target DNA sites.

We next evaluated the effect of AROS and DBC1 knockdown on the activation of an *hsp* promoter reporter. HeLa cells expressing a stable *hsp70.1* promoter-luciferase reporter were transfected with siRNA to HSF1, AROS, DBC1, or a non-targeting (NT) siRNA control. Cells were treated with either DMSO (mock) or celastrol (5 µM) 24 hours post-transfection and luciferase activity was measured 24 hours post-treatment. We found that knockdown of AROS resulted in a 2-fold reduction of celastrol-induced luciferase activity compared to the NT siRNA control (Fig. 6E). This result is similar to the result produced with HSF1 siRNA treatment. Conversely, DBC1 knockdown resulted in an approximate 2.3-fold increase in celastrol-induced luciferase activity compared to the non-targeting siRNA control (Fig. 6E). This data provides further support that AROS and DBC1 positively and negatively impact expression from the *hsp70* promoter, respectively.

## Discussion

### HSF1 and Post-translational Modification

HSF1, the master regulator of genes involved in protein quality control, is highly post-translationally modified [1]. These modifications are hypothesized to allow fine-tuning of the transcriptional activity of HSF1 in order to respond to the precise needs of the cell. We have previously shown that HSF1 is acetylated and SIRT1 activates HSF1 through deacetylation at a key lysine residue within the DNA binding domain, thereby promoting DNA binding [15]. With this study, we have expanded our analysis of the regulation of HSF1 by SIRT1 and find that AROS and DBC1, two SIRT1 modulators, can regulate HSF1’s acetylation status and thus control the HSR.

### SIRT1 and the HSR

Prior work by us and others has established a connection between SIRT1 and the HSR in mammalian tissue culture cells, mice, and *C. elegans* [*15*], [23], [*30*]. We have shown that SIRT1 prolongs HSF1 DNA binding, thus serving to enhance the HSR by increasing HS-induced chaperone expression [15]. The connection between SIRT1 and the HSR has been further established with an α-synuclein mouse model [23]. In these mice, SIRT1 was shown to deacetylate HSF1 and increase HSP70 levels in the brain, leading to α-synuclein aggregate suppression and an overall greater survival [23]. We have also recently shown that the *C. elegans* SIRT1 homolog Sir2.1 is linked to the HSR [30]. Caloric restriction was found to synergize with HS to induce *hsp70* gene expression in the worm, and this effect was dependent on Sir2.1 [30]. Our finding here that DBC1 and AROS, two modulators of SIRT1 activity, can regulate the HSR provides yet further support for a role of SIRT1 in the HSR.

### The Regulation of SIRT1 during Heat Shock

We sought to discover how HS may regulate SIRT1 activity. We first tested whether SIRT1 may be regulated at the transcription or translation level during HS. However, we did not find major changes in SIRT1 abundance throughout a HS timecourse. SIRT1 is a NAD^+^ binding protein and has been shown to be activated by an increase in the cellular NAD^+^/NADH ratio [17]. Previous studies have indicated that a 10-fold change in NAD+ concentration was required to affect Sir2 activity [31]. However, a decrease in NADH, a competitive inhibitor of SIRT1 that is present at much lower levels in the cell compared to NAD+, may have a greater impact on SIRT1 activity. Caloric restriction studies in yeast have shown that Sir2 activity is regulated by a reduction of NADH resulting in an increase in the NAD+/NADH ratio [18], [32]. We demonstrate in this study that cellular NADH levels decrease upon HS resulting in an increase in the NAD+/NADH ratio and therefore this may be one mechanism by which HS regulates HSF1 activity. These results suggest that the HS-induced increase in the NAD^+^/NADH ratio may be an important factor in SIRT1 regulation of the HSR.

While SIRT1 is not a DNA binding protein, it can be recruited to promoters through association with other DNA binding proteins. For instance, BCL11A, a zinc finger transcription factor that functions as a myeloid and B-cell proto-oncogene, has been shown to recruit SIRT1 to a promoter template [33]. Furthermore, SIRT1 converges to the same DNA regulatory elements as its transcription factor substrate CLOCK, indicating that these proteins operate on circadian promoters in a chromatin regulatory complex [34]. We sought to investigate whether SIRT1 was recruited to the *hsp70* promoter with similar kinetics as HSF1 upon HS and found that it is. Therefore, SIRT1 may be in a chromatin-bound complex together with HSF1 on target promoters, leading to enhanced HSF1 activity.

### AROS and DBC1 Regulate HSF1

AROS and DBC1 are two regulators of SIRT1 activity. DBC1 has been shown to form a stable complex with SIRT1 by binding to its catalytic domain and inhibiting its activity by blocking substrate access [20], [21]. While AROS regulation of SIRT1 has been comparatively understudied, it is a SIRT1 binding partner that positively regulates SIRT1 deacetylase activity [22]. AROS and DBC1 were first characterized to affect SIRT1 regulation of p53-mediated apoptosis through alteration of the p53 acetylation status. DBC1 has also recently been characterized to regulate the acetylation status of the nuclear receptor PPARγ [35].

We show here that the overexpression and knockdown of AROS and DBC1 have a striking impact on *hsp70* transcription. Overexpression of DBC1 results in a decrease in *hsp70* transcription, while knockdown with DBC1 siRNA results in an increase in HSR induction. Conversely, overexpression of AROS results in an increase in *hsp70* transcription, while knockdown with AROS siRNA results in a decrease in HSR induction. In an acetylation assay, AROS is able to completely deacetylate HSF1 in a manner similar to SIRT1, while DBC1 increases HSF1 acetylation. Given that AROS and DBC1 were found to regulate HSF1 acetylation, and HSF1 acetylation can affect DNA binding ability, we sought to investigate whether AROS and DBC1 expression could affect HSF1 DNA binding. As expected, we found that overexpression of AROS led to an increase in HSF1 recruitment to the *hsp70* promoter and that, conversely, overexpression of DBC1 led to a decrease in HSF1 recruitment to the promoter. Thus, we have now added HSF1 to the list of SIRT1 target proteins that are modulated at the acetylation status level by AROS and DBC1. This work expands our knowledge of the control of the HSR and provides AROS and DBC1 as new therapeutic targets for modulating this response.

## Methods

### Cell Culture and Heat Shock

HeLa, HEK293, and HeLa *hsp70.1* promoter-luciferase reporter cells [29] were utilized in this study. All cell lines were cultured in DMEM media (CellGro cat#15-017-CV) supplemented with 10% fetal bovine serum (GIBCO cat#10437-028) and 1% *Pen*-*Strep*-Glutamine (CellGro cat#30-0090CI) at 37°C with 5% CO_2_. HS was induced as previously described [15]. Briefly, plates were wrapped in parafilm and submerged in a water bath set to 42°C for the designated times prior to collection with 1X PBS.

### Plasmids

Plasmids used in this study include expression vectors for SIRT-MYC [36], p300 [37], DBC1-HA [20], AROS [22], Flag-HSF1 and Myc-HSF1 [38], [39].

### NAD^+^/NADH Quantification

HeLa cells were exposed to control conditions or a 2 hour HS in a 42°C water bath. The cells were collected in 1X PBS and NAD^+^/NADH extracted using the FluoroNAD**™** Fluorescent NAD/NADH Detection Kit by Cell Technology, Inc (cat#FLNADH 100-2), according to the manufacturer’s instructions. The concentration readings were applied to a NADH standard curve and the NAD^+^/NADH ratio was determined.

### Chromatin Immunoprecipitation

HEK293 cells were fixed in 1% formaldehyde and neutralized in 125 mM glycine. The cells were collected in 1X PBS, lysed in Cell Lysis Buffer (5 mM PIPES pH 8.0, 85 mM KCl, 0.5% NP40) with the addition of Halt™ Protease Inhibitors (Thermo Scientific cat#78430), and then centrifuged, with the resulting pellet resuspended in Nuclei Lysis Buffer (50 mM Tris-HCl pH 8.1, 10 mM EDTA, 1% SDS) with the addition of Halt™ Protease Inhibitors. Chromatin shearing was performed for 45 minutes, cycling on and off for 30 seconds each using the Diagenode Bioruptor 300. Immunoselection and immunoprecipitation were performed essentially as previously described [40] using 5 µL of HSF1 (Enzo cat#ADI-SPA-901), SIRT1 (Millipore cat #07-131), or IgG (Cell Signaling Technology cat#2729S) antibody. The DNA was purified using phenol/chloroform/isoamyl alcohol (25∶24:1) by standard procedure. qPCR was performed on the ChIP DNA with ABI’s Step One Plus Real-time PCR system using BioRad’s iTaq™ Fast SYBR**®** Green Supermix with ROX (cat#172-5101) according to manufacturer’s protocol using primers to the *hsp70* promoter-proximal HSE site, an upstream *hsp70* promoter site, and the *gapdh* promoter (Table 1). Statistical data analysis and determination of relative fold increase from control samples without HS was performed according to standard protocol [41].

**Table 1:** List of PCR primers used in this study.

Gene name	Sequence*	Amplicon (bp)
*aros*	F: 5′- GAAGGCAATTCAGGCCCAGAAACT- 3′	131
	R: 5′- TCGTCCTGGTCAGAAACTTCAGGT- 3′	
*dbc1*	Primer mix from GeneCopoeia, Inc.	Not provided
	Cat # HQP015872 (KIAA1967)	
*gapdh*	F: 5′- CCACTCCTCCACTTTGAC - 3′	102
	R: 5′- ACCCTGTTGCTGTAGCCA - 3′	
*gapdh* promoter^1^	F: 5′- TACTAGCGGTTTTACGGGCG - 3′	166
	R: 5′- TCGAACAGGAGGAGCAGAGAGCGA - 3′	
*hsp27* 2	F: 5′- CAAGTTTCCTCCTCCCTGTC - 3′	156
	R: 5′- GGCAGTCTCATCGGATTTTG - 3′	
*hsp70* 3	F: 5′- AGAGCGGAGCCGACAGAG - 3′	110
	R: 5′- CACCTTGCCGTGTTGGAAC - 3′	
*hsp70* promoter 1^3^	F: 5′- GGCGAAACCCCTGGAATATTCCCGA - 3′	191
(HSE site)	F: 5′- AGCCTTGGGACAACGGGAG - 3′	
*hsp70* promoter 2	F: 5′- CCTCCCGAGGAGCTGGGACT - 3′	134
(NS site)	R: 5′- CGAGGCGGGCGGATCACTTA - 3′	
*hsp90* 4	F: 5′- GGCAGTCAAGCACTTTTCTGTAG - 3′	199
	R: 5′- GTCAACCACACCACGGATAAA - 3′	
*sirt1* 3	F: 5′- TCCTGGACAATTCGAGCCATCTCT - 3′	103
	R: 5′- TTCCAGCGTGTCTATGTTCTGGGT - 3′	

* F: forward, R: reverse.

1 Primers from Folz *et al. Am. J. Respir. Cell Mol. Biol.* 2008 **39**:2, 243.

2 Primers from Yao *et al. Journal of Biomedical Science* 2010 **17**:30.

3 Primers from Westerheide *et al. Science* 2009 **323,** 1063.

4 Primers from McLean *et al. Biochemical and Biophysical Research Communications* 2006 **351**:3.

### Transient Transfection and siRNA Knockdown

Transfections were performed with Polyfect® Transfection Reagent (Qiagen cat#301107) according to the manufacturer’s protocol. HeLa *hsp70.1* promoter-luciferase reporter and HEK293 cells were transfected with DharmaFECT transfection reagent (Thermo) according to the manufacturer’s protocol, using 50 nM of Dharmacon SmartPool DBC1, AROS, HSF1, or non-targeting (NT) control siRNA. RNA was isolated using Trizol 48 hours after transfection. Knockdown was confirmed via qRT-PCR.

### Cell Viability Assay

Cell viability was measured 48 hours after treatment with siRNA or expression plasmids using PrestoBlue (Life Technologies Ltd. cat#A13262) according to the manufacturer’s instructions. Briefly, HEK293 cells were seeded in a 96 well plate and transfected as described above. After 48 hours of treatment, PrestoBlue was added to each well at a final concentration of 10%. After a 10 minute incubation at 37°C, total fluorescence was measured at excitation 525+/−20 nm and emission 590+/−35 nm.

### HSF1 Acetylation Assay

HEK293 cells were transfected with expression plasmids Flag-HSF1 and p300, and either SIRT1-MYC, DBC1-HA, or AROS. Cell lysates were subjected to immunoprecipitation with Flag beads, and acetylated HSF1 was detected by Western blotting with an antibody that recognizes acetylated lysines (Cell Signaling Technology cat#9441).

### Luciferase Assay

HeLa *hsp70.1* promoter-luciferase reporter cells were seeded in a 96 well plate at a density of 7.5×10^3^ cells per well. All transfections were performed 24 hours after plating. 24 hours post-transfection cells were treated with or without 5 µM celastrol. Luciferase assays were performed 24 hours post-treatment with Promega’s Bright-Glo Luciferase Assay (cat#E2620) according to manufacturer’s instructions and as previously described [42].

### Quantitative RT-PCR

qRT-PCR was performed to quantify mRNA levels for *aros*, *dbc1*, *hsp27*, *hsp70, hsp90*, *sirt1,* and *gapdh* using gene-specific primers (Table 1). Cells were collected in PBS and RNA was extracted using Trizol per standard protocol. RNA was reverse transcribed using Applied Biosystem’s High Capacity cDNA Reverse Transcription Kit (cat#4368814) according to the manufacturer’s protocol. The samples were diluted to 50 ng/µL and used as a template for qRT-PCR. qRT-PCR was performed with Applied Biosystem’s Step One Plus Real-time PCR system using BioRad’s iTaq™ Fast SYBR**®** Green Supermix with ROX (cat#172-5101) according to manufacturer’s protocol. Statistical data analysis and determination of relative fold increase from control samples was performed according to standard calculations [41].

### Immunoblotting Analysis

The cells were collected in 1X PBS and then extracted using M-PER (Thermo Scientific cat#78503) with the addition of Halt™ Protease Inhibitors (Thermo Scientific cat#78430). Protein was quantified with Biorad Protein Assay (cat#500-0006) and 20 µg of protein was run on 10% SDS-PAGE gels. Gels were stained with 1% Ponceau in 10% glacial acetic acid for visualization of normalized protein levels before being blotted to nitrocellulose and immunostained with primary antibody in 5% milk. Primary antibodies used include ACTIN (Santa Cruz cat#sc-1616-r), AROS (Santa Cruz cat#sc-86210), β-tubulin (Cell Technology cat#2128), DBC1 (Abcam cat#ab70242), HA (Convance cat# MMS-101P), c-MYC (Sigma cat#M4439), HSF1 (Assay Design cat#SPA-950), SIRT1 (Abcam cat#ab 32441), Acetylated-Lysine (Cell Signaling Technology cat#9441). HRP-conjugated secondary antibodies were from Millipore (cat#12-349 and 12-348) and Jackson ImmunoResearch (cat#112-035-062). ECL Plus Western Blotting Detection System (Amersham™ cat#RPN2132) was used to incubate blots prior to film exposure (Kodak ClinicSelect Blue X-ray film cat#604-1768).
